# Supplementary material for: First-in-human evaluation of real-time pixel-level AI-assisted anatomical segmentation in neurosurgery: pituitary surgery as an exemplar
Source: Pituitary. 2026 Oct 3;29(5):163. doi: 10.1007/s11102-026-01762-x (PMC13634013; doi:10.1007/s11102-026-01762-x)
Supplement: Supplementary file 2 [file 11102_2026_1762_MOESM1_ESM.docx]

**Supplementary Material**

Supplementary Material 1: Comparative analysis with retrospective cohort. Given the small prospective sample size, no formal statistical hypothesis testing was performed. OSATS: Objective Structured Assessment of Technical Skills

| **Outcome measure** | **This study**  **(8 recruited)** | **This study**  **(6 CVAI-deployed)** | **Retrospective cohort**  **(Khan et al, n=63)[32]** |
| --- | --- | --- | --- |
| **Surgical performance** | | | |
| Mean sellar phase OSATS (/30) | 27.1 ± 1 | 27.2 ± 1 | 28.6 ± 0.8 |
| **Surgical outcomes** | | | |
| Intra-operative vascular injury | 0/8 (0%) | 0/6 (0%) | 0/63 (0%) |
| Visual decline | 0/8 (0%) | 0/6 (0%) | 0/63 (0%) |
| New anterior pituitary hormone deficiency requiring supplementation | 3/8 (38%) | 2/6 (33%) | 26/63 (41%) |
| New posterior pituitary deficiency requiring supplementation | 0/8 (0%) | 0/6 (0%) | 2/63 (3.2%) |
| Hyponatraemia / dysnatraemia requiring treatment | 2/8 (25%) | 1/6 (17%) | 7/63 (11%) |
| Postoperative CSF leak | 0/8 (0%) | 0/6 (0%) | 4/63 (6.4%) |
| Length of stay, median (days) | 4 | 4 | 4 |
